# Nuclear locus divergence at the early stages of speciation in the Orchard Oriole complex

**DOI:** 10.1002/ece3.2168

**Published:** 2016-05-30

**Authors:** Rachel J. Sturge, M. Nandadevi Cortés‐Rodríguez, Octavio R. Rojas‐Soto, Kevin E. Omland

**Affiliations:** ^1^ Department of Biological SciencesUniversity of MarylandBaltimore County1000 Hilltop CircleBaltimoreMaryland21052; ^2^ Red de Biología EvolutivaInstituto de Ecología (INECOL)Carretera antigua a Coatepec 351Xalapa91070Mexico

**Keywords:** *Icterus fuertesi*, *Icterus spurius*, mtDNA paraphyly, Orchard Oriole complex, phylogeography, recent speciation

## Abstract

As two lineages diverge from one another, mitochondrial DNA should evolve fixed differences more rapidly than nuclear DNA due to its smaller effective population size and faster mutation rate. As a consequence, molecular systematists have focused on the criteria of reciprocal monophyly in mitochondrial DNA for delimiting species boundaries. However, mitochondrial gene trees do not necessarily reflect the evolutionary history of the taxa in question, and even mitochondrial loci are not expected to be reciprocally monophyletic when the speciation event happened very recently. The goal of this study was to examine mitochondrial paraphyly within the Orchard Oriole complex, which is composed of *Icterus spurius* (Orchard Oriole) and *Icterus fuertesi* (Fuertes' Oriole). We increased the geographic sampling, added four nuclear loci, and used a range of population genetic and coalescent methods to examine the divergence between the taxa. With increased taxon sampling, we found evidence of clear structure between the taxa for mitochondrial DNA. However, nuclear loci showed little evidence of population structure, indicating a very recent divergence between *I*. *spurius* and *I. fuertesi*. Another goal was to examine the genetic variation within each taxon to look for evidence of a past founder event within the *I. fuertesi* lineage. Based on the high amounts of genetic variation for all nuclear loci, we found no evidence of such an event – thus, we found no support for the possible founding of *I. fuertesi* through a change in migratory behavior, followed by peripheral isolates speciation. Our results demonstrate that these two taxa are in the earliest stages of speciation, at a point when they have fixed differences in plumage color that are not reflected in monophyly of the mitochondrial or nuclear DNA markers in this study. This very recent divergence makes them ideal for continued studies of species boundaries and the earliest stages of speciation.

## Introduction

For the last 25 years, neutral molecular markers, especially mitochondrial DNA (mtDNA), have been used to examine closely related species – elucidating their evolutionary histories and looking for evidence of gene flow. Studies of closely related taxa often address species delimitation by looking for reciprocal monophyly in mitochondrial genes (Moritz 1994; Zink and McKitrick 1995; Funk and Omland 2003; Hebert et al. 2004; Zink and Barrowclough 2008). Mitochondrial DNA has two key advantages that make it useful in this regard: (1) mtDNA has a rapid mutation rate, allowing for increased chances of accumulating variation; (2) mtDNA also has faster sorting (genetic drift) due to its maternal inheritance and generally lower effective population sizes (ESSs; Avise 1994; Zink and Barrowclough 2008).

However, there are many examples where species delimitation based solely on mtDNA would disagree with boundaries delimited using other characters (Baker et al. 2003; Funk and Omland 2003; Olsson et al. 2005; Omland et al. 2006; Knowles and Carstens 2007; Joseph and Omland 2009). There are several possible reasons for this disagreement. For instance, individual gene trees of closely related taxa can have unpredictable amounts of variation due to stochasticity in mutation, genetic drift, and sampling error (Rosenberg and Nordborg 2002; Knowles and Carstens 2007). A major factor that limits the utility of mtDNA is that because it is haploid and maternally inherited, different mitochondrial genes are inherited as a group, sharing a single evolutionary history that may or may not accurately represent the evolutionary history of the species of interest (Knowles and Carstens 2007). A second problem with the approach mentioned above is its focus on one criterion – the presence of reciprocal monophyly. Recent studies have shown that a substantial time lag exists between species divergence and the development of reciprocal monophyly in gene trees (Hey 2006; Omland et al. 2006; Knowles and Carstens 2007). Neutral genetic changes can diverge slower than traits under selective pressure, which may result in genetically indistinct taxa with fixed differences in phenotypes that play a role in fitness or sexual selection (e.g., Joseph and Omland 2009; Ross 2014). Species delimited by genetic markers thus can disagree with those delimited using other characteristics, such as morphology. Thus, recently diverged species will most likely not have any fixed differences in their mtDNA due to this time lag, as is evidenced by the multiple examples of known gene tree paraphyly in Animalia (see the following papers for a detailed explanation and for examples: Edwards and Beerli 2000; Funk and Omland 2003; Joseph and Omland 2009) and the many well‐recognized species that lack fixed mtDNA differences (Ross 2014). Our study focuses on a pair of taxa that represent a well‐documented case of mitochondrial paraphyly: the Orchard Oriole complex.

**Figure 1:**
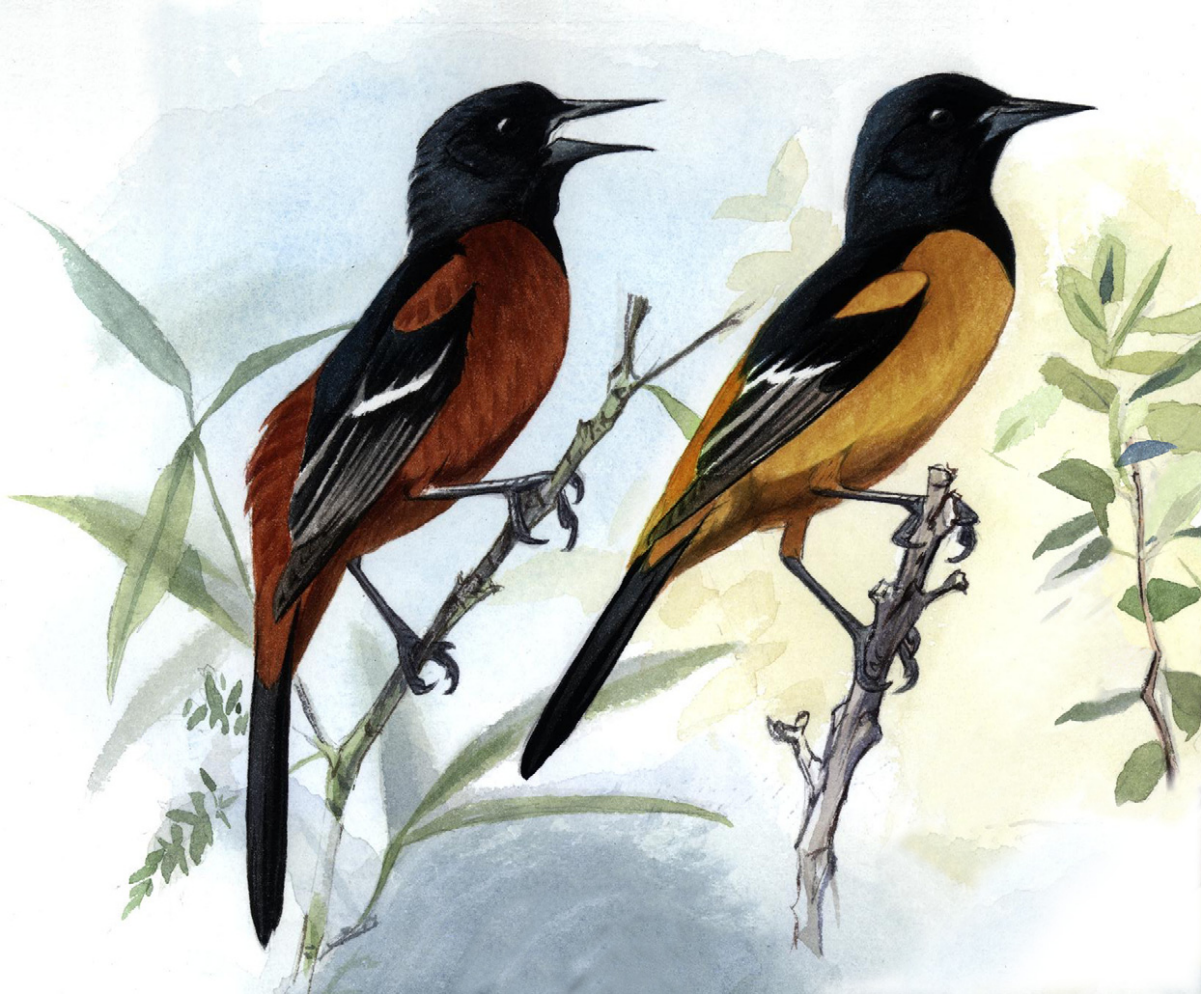
Adult male *Icterus spurius* (left) and *I. fuertesi* (right). Adult females and yearling males of the two taxa are predominantly olive yellow; the two taxa cannot be distinguished in these plumages. Painting by J. C. Anderton.

The Orchard Oriole complex is comprised of two taxa: *Icterus spurius* (Orchard Oriole), which breeds from southeastern Canada, across the eastern United States and into north‐central Mexico, and *Icterus fuertesi* (Fuertes' Oriole), which breeds in a geographically restricted region along the coastal lowlands of southern Tamaulipas and Veracruz, Mexico (Howell and Webb 1995). These two taxa are considered separate species by the IOC World Bird List (Gill and Donsker 2015; also Navarro and Peterson 2004). Clements World Checklist (e.g., Clements 2007) formerly considered them separate species, but they were recently reclassified as subspecies within *I. spurius,* which is also followed by the American Ornithologist's Union (Chesser et al. 2015). In this article, we follow the taxonomy of IOC (Gill and Donsker 2015). The controversy regarding the separation of Orchard Oriole and Fuertes' Oriole into different species is based on a variety of studies carried out over the last 10 years. The two taxa differ in a number of ways. Adult males show a fixed difference in color (Fig. 1), with no overlap in color variation when their plumage is analyzed through spectrophotometric comparisons (Hofmann et al. 2007; Kiere et al. 2007). Adult males of the two taxa are easily distinguished visually in the field (pers. obs.). They also differ in migratory behavior, as *I. spurius* migrates whereas *I. fuertesi* is a short‐distance migrant or partial migrant (Jaramillo and Burke 1999; Tobón‐Sampedro and Rojas‐Soto 2015). These taxa breed at different latitudes, which has resulted in bioclimatic niche differentiation for their breeding distributions (Martin and Omland 2011). Lastly, studies of their vocalizations have found no evidence of song differentiation (Hagemeyer et al. 2012), yet a call that appears to play a role in territory defense differs significantly in a number of different measures of frequency, duration, and amplitude (Sturge et al. 2016).

In addition, Baker et al. (2003) sampled these two taxa across their ranges and sequenced two regions of mtDNA (cytochrome *b* and control region). They found that the two species were paraphyletic in their mtDNA with intermixed haplotypes (haplotypes that are shared and/or show paraphyletic relationships with another species; Joseph and Omland 2009). *Icterus spurius* and *I. fuertesi* show both these patterns of intermixing in their mtDNA. However, the frequencies of different mitochondrial haplotypes differ significantly, indicating little or no mitochondrial gene flow between the two taxa (*F*_ST_ = 0.608, *P* < 0.00001, Baker et al. 2003). Baker et al. (2003) suggested that retained ancestral polymorphisms could be sufficient to explain this mitochondrial paraphyly between the two taxa, but they did not test it with rigorous statistical analyses (i.e., coalescent methods using multiple loci).

The mitochondrial paraphyly in the Orchard Oriole complex has two possible explanations, as with other cases. First, the split between the two taxa could have happened so recently that the haplotype variation within each of these taxa has not yet coalesced; thus, they could share haplotypes due to retained ancestral polymorphisms. Second, it is possible that gene flow has been occurring since the two taxa diverged, which could explain the observed patterns of haplotype and lineage sharing. In avian species, hybridization and gene introgression are common even among more distantly related species (Peters et al. 2007; Price 2008; Rheindt and Edwards 2011), and species divergence can occur even in the face of ongoing gene flow (Hey 2006).

**Figure 2:**
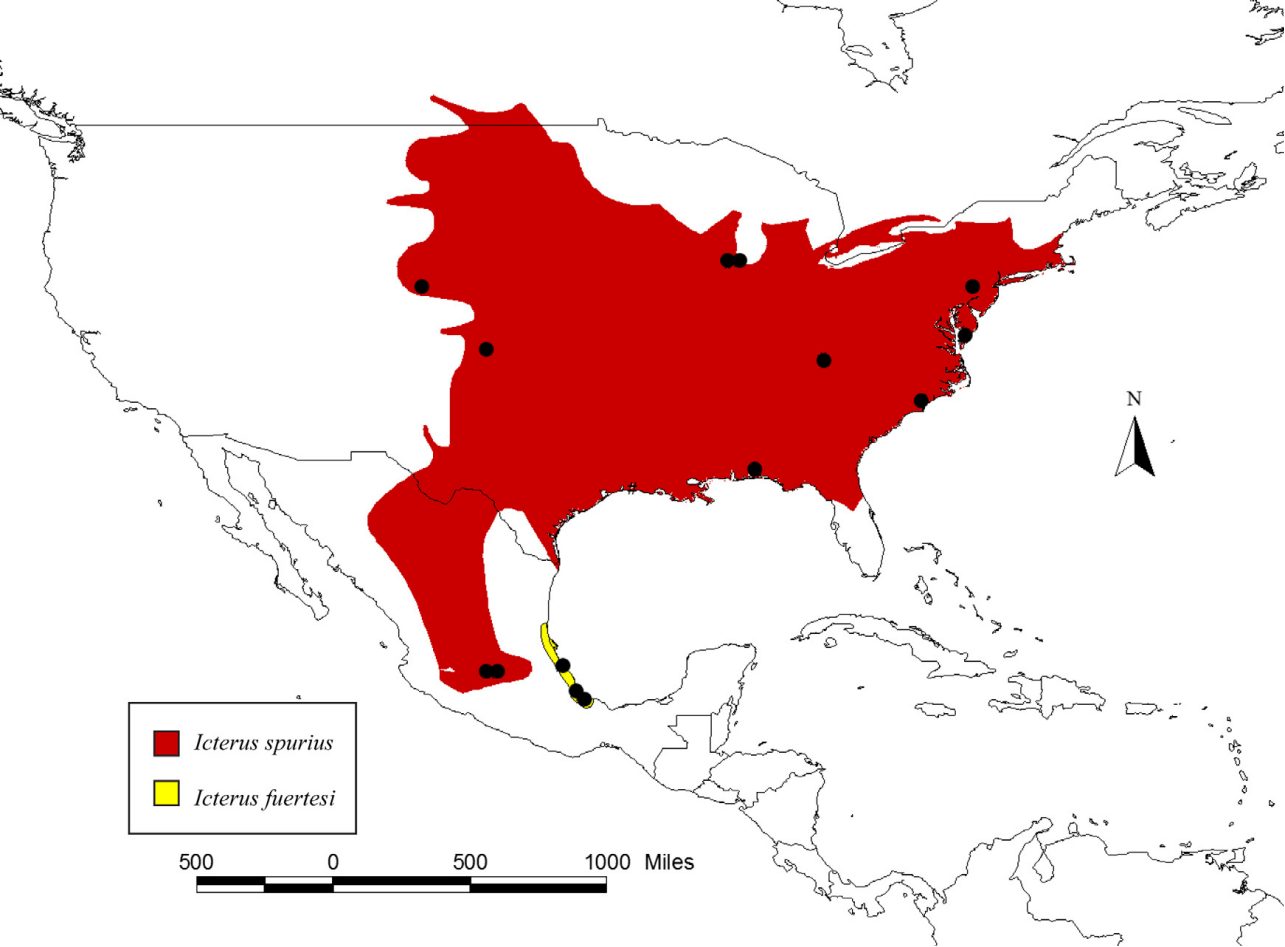
Map showing sampling locations (black dots) for *Icterus spurius* and *I. fuertesi*. All the dots for *I. fuertesi* represent localities where we collected multiple samples. This map also shows the breeding ranges for both species. The map was built using ArcView 3.2 (Environmental Systems Research Institute, Redlands, CA) and the distributional ranges from Nature Serve (www.natureserve.org) and by adding each locality point where the samples were collected.

Additionally, the results from Baker et al. (2003) suggested that the divergence that led to the formation of these two taxa could have been the result of a founder event within the lineage leading to *I. fuertesi*. This was based on two mitochondrial genes (cytochrome *b* and control region), for which *I. fuertesi* showed lower haplotype diversity than *I. spurius* – which is the predicted pattern if the lineage leading to *I. fuertesi* was founded from a few individuals whose genetic diversity only represented a portion of the available diversity within the ancestral population (Harrison 1991; Baker et al. 2003; Funk and Omland 2003). The two taxa within the Orchard Oriole complex exhibit different migratory behavior, as mentioned above – with *I. spurius* migrating long distances while *I. fuertesi* is a short‐distance migrant that overwinters in the southern portion of its breeding range, where it likely overlaps with overwintering *I. spurius* (Howell and Webb 1995; Jaramillo and Burke 1999; Kondo and Omland 2007; Tobón‐Sampedro and Rojas‐Soto 2015). As migratory behavior is highly labile in orioles (Winker 2000; Kondo and Omland 2007), a loss or decrease in migratory behavior within a subset of the ancestral population could have resulted in the founding of the current breeding grounds of *I. fuertesi*, followed by peripheral isolates speciation in allopatry (Mayr 1942, 1963; West‐Eberhard 2005). Examining Figure 2, *I. fuertesi* looks like it could be a classic example of peripheral isolates speciation based on its much smaller breeding range, which is geographically isolated from the closest *I. spurius* breeding populations by the Sierra Madre Oriental Mountains. However, the lack of genetic variation within *I. fuertesi* reported by Baker et al. (2003) could also have resulted from differences in sampling effort – there were roughly twice as many *I. spurius* individuals as *I. fuertesi* included in their study.

We addressed these questions regarding the mitochondrial paraphyly between these taxa, and the potential founder event involving *I. fuertesi* by sequencing multiple loci (four nuclear introns) and through an increase in sample size, adding nine *I. fuertesi* samples. We sequenced multiple nuclear loci to obtain a broader picture of the genomic divergence between these two taxa. Using multiple loci, we attempted to avoided either stochastic or deterministic processes that might have only affected a part of the genome or one type of marker (e.g., mtDNA; Edwards and Beerli 2000; Peters et al. 2008). We also used population coalescent methods, including IM (isolation with migration, Hey and Nielsen 2004; Hey 2006) to examine polyphyly and shared haplotypes to see whether it was possible to differentiate between retained ancestral polymorphisms and gene flow as possible explanations for these patterns. Our goals were to address the following questions: (1) Do nuclear loci also support very recent divergence, or does mtDNA reflect recent introgression between two well‐diverged lineages? (2) What is the nature of the nuclear intron variation between these two taxa? Do they support two distinct groups? (3) Was divergence the result of a founder event and peripheral isolates speciation? (4) Is it possible to determine whether these two taxa are currently exchanging genes?

## Methods

### DNA sequencing

In this study, we had 32 samples already available to us and we collected nine additional *I. fuertesi* samples from multiple localities in Veracruz, Mexico, to increase our sample size. Not all of the old samples would amplify; as a result, we included samples from 25 *I. spurius* and 14 *I. fuertesi* from across their breeding ranges (Fig. 2, Table S1). For each individual, we sequenced a total of five loci: four nuclear introns and one mitochondrial gene. The four nuclear introns were located on four different chromosomes of the zebra finch genome; three were autosomal loci (GADPH11 397 b.p., TGFB2 574 b.p. and RDP2 298 b.p.) and one was on the Z sex chromosome (SLC9 429 b.p.). The fifth locus was the mitochondrial control region (CR – domain I; 344 b.p.). Accession numbers on GenBank for the genes we sequenced are as follows: GADPH11 (KU903008–KU903079), C.R. (KU903080–KU903118), SLC9 (KU903119–KU903152), RDP2 (KU903153–KU903221), and TGFB2 (KU903222–KU903282). For additional information about the five loci, see Table 1.

**Table 1:** Information on the loci (one mitochondrial locus, three autosomal introns, and one Z‐linked intron) used in this study

Locus	Chromosome	Length	*N*		Annealing temperature (°C)	Primer source
			*Icterus spurius*	*Icterus fuertesi*		
CR‐Domain I	Mitochondrial	344	25	16	52	Kondo et al. (2004)
GADPH11	1	397	48	28	55	Primmer et al. (2002)
TGFB2	2	574	44	28	65	Bureš et al. (2002)
RDP2	12	298	44	28	56	Waltari and Edwards (2002)
SLC9	Z chromosome	429	35	21	56	Bandelt et al. (1999)

Chromosome locations were determined using BLAST searches of a reference genome (Zebra Finch). The number of alleles for each taxon are given as *N*.

We extracted DNA from muscle tissue of all samples using a DNeasy Blood and Tissue Kit (QIAGEN, Valencia, CA). PCRs were then performed on each locus using a Gene Amp PCR System 9700 (Applied Biosystems, Foster City, CA), and we verified our product using 1% agarose gels with ethidium bromide. We cleaned PCR product using QIAquick PCR Purification Kits (QIAGEN). The amplified loci were sequenced using ABI's BigDye v. 3 Terminator Cycle Sequencing Kit on the Gene Amp PCR System 2400 (Applied Biosystems). We used EDTA‐ethanol precipitation to remove excess dye terminators. Chromatograms for each sequence were produced using the University of Maryland, Baltimore County's ABI3100 DNA Sequencer (Applied Biosystems). We edited and aligned the chromatograms using Sequencher 4.1 (Gene Codes Corporation, Ann Arbor, MI).

Individuals within our dataset that contained more than one polymorphic site within a locus were phased using PHASE 2.1.1 (Stephens et al. 2001; Stephens and Scheet 2005). We set the burn‐in rate to 1000 and the thinning interval to 1. Each run included 10,000 iterations, and we repeated each analysis ten times with different random numbers as the starting seed. We selected the haplotype states suggested by the runs with the highest log‐likelihood values and included all haplotypes that resolved at a probability of at least 0.5 (for justification of this approach and cutoff value, see Jacobsen and Omland 2012). All of the SNP phasing in this study met this cutoff, so no further action was necessary. TOPALi 2.5 (Milne et al. 2004) was used to look for evidence of intralocus recombination – none of our loci had a significant DSS peak and so all were included in our dataset. We constructed and edited haplotype networks within NETWORK v. 4.6.1.1 (Bandelt et al. 1999) using the median joining algorithm.

### Population statistics and structure

The number of haplotypes (*h*), the haplotype diversity (Hd), the nucleotide diversity (*π*), the average number of nucleotide differences per site (*p*), and the number of nucleotide polymorphisms (*θ*) were all calculated using DnaSP (Rozas et al. 2003; for results, see Table 2). We also used DnaSP to calculate Tajima's *D* (Tajima 1989), Fu and Li's F* (Fu and Li 1993), Ramos‐Onsins & Roza's neutrality state (*R*_2_, Ramos‐Onsins and Rozas 2002), and mismatch distributions (with associated Tau and Theta values). To look for significant population structure within the nuclear loci, we used PGDSpider to produce a dataset that could be analyzed within Structure by assigning each haplotype a unique number (Lischer and Excoffier 2012). One of the assumptions of Structure is that the different loci included are unlinked. As mentioned previously, our loci were located on different chromosomes of the Zebra Finch genome, so our approach should not violate this assumption. Note that this method does not consider the similarity between different haplotypes when it assigns numerical designations. We analyzed our data using Structure v. 2.3.4 (Pritchard et al. 2000; Hubisz et al. 2009). We used the admixture model with correlated allele frequencies and the LOCPRIOR flag activated, and set both the Dirichlet parameter for degree of admixture (*α*) and the allelic frequency parameter (*k*) to be inferred from the dataset, rather than being fixed. All runs had a burn‐in of 50,000, followed by 250,000 iterations to collect data to estimate posterior probabilities. We tested *K* values from 1 to 10, and replicated each run 20 times for all *K* values.

**Table 2:** Estimates of the number of haplotypes (*h*), the haplotype diversity (Hd), and the nucleotide diversity (*π*) for the loci included in this study

Locus	*Icterus spurius* (Orchard Oriole)			*Icterus fuertesi* (Fuertes' Oriole)		
	*h*	Hd	*π*	*H*	Hd	*π*
CR‐ mtDNA	9	**0.597**	0.00276	3	0.242	0.01410
GADPH11	14	**0.840**	0.00531	11	0.807	0.00655
TGFB2	16	0.862	0.00509	12	**0.889**	0.00441
RDP2	12	**0.852**	0.00682	7	0.794	0.00507
SLC9	10	0.724	0.00285	8	**0.886**	0.00433

For each locus, the highest haplotype diversity between the two species is shown in bold.

We used Arelquin v3 3.5.1.2 (Excoffier and Lischer 2010) to run AMOVAs on each locus to test for significant population structure within and between the two taxa (Schneider et al. 2000; Excoffier and Lischer 2010). We calculated Φ_ST_ values in Arelquin as well**,** which are analogous to Wright's *F*_ST_ values, but take into account the number of mutations between each haplotype within the sample (Excoffier and Lischer 2010).

### Coalescent analyses

To determine whether or not we could estimate how much of the shared DNA variation between these two taxa is due to retained ancestral polymorphisms versus gene flow (either historic or ongoing), we analyzed mtDNA, nuclear DNA, and the combined mtDNA and nuclear DNA datasets using both the programs IM and IMa (isolation with migration, Hey 2006, 2007). IM estimates seven demographic parameters: ESSs of the ancestral (*θ*_A_) and daughter populations (*θ*_1_ and *θ*_2_), migration rates between the two daughter populations (*m*_1_ and *m*_2_), and the time the two daughter populations diverged from each other (*t*), and the contribution of the ancestral population to each daughter population (splitting parameter, *s*) (Hey and Nielsen 2004). We used the infinite sites model and set the inheritance scalars based on the modes of inheritance for the loci (autosomal were set to 1, Z linked to 0.75 and mitochondrial to 0.25). In these analyses, we included multiple heated chains (30 chains) and examined the ESS estimates to insure the Markov chains were mixing appropriately (Hey and Nielsen 2004). We also included 1,000,000 generations and sampled every 100 generations so we could monitor output. We completed the run multiple times, using different starting seeds for each run. We also varied the following parameters in an effort to reach convergence: the maximum migration rates for both populations (*m*_1_ and *m*_2_), the maximum time of population splitting (*t*), the duration of the burn (*b*) and the duration of the run (*l*), the heating parameters (*g*_1_ and *g*_2_), the number of chains (*n*) and the number of chain swap attempts per step (*k*), and the estimates of maximum ancestral and daughter population sizes (*q*_A_, *q*_1_ *,* and *q*_2_).

## Results

**Figure 3:**
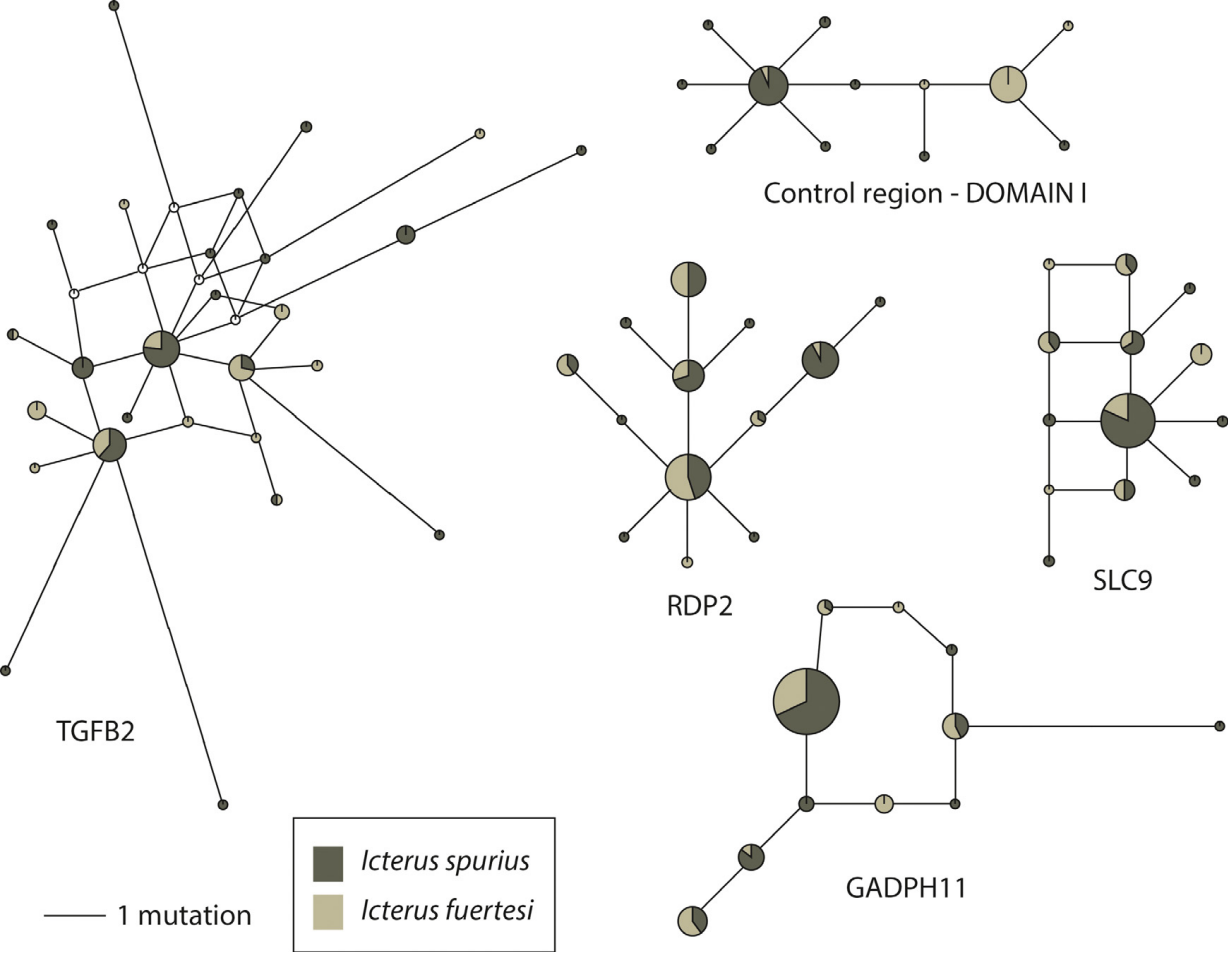
Haplotype networks for four nuclear introns (three autosomal and one Z‐linked) show extensive haplotype sharing between *Icterus spurius* and *I. fuertesi*. In contrast, the haplotype network for the mitochondrial control region shows very little intermixing and only one shared haplotype between the two taxa. Circle sizes correspond to sample sizes of each allele.

Both taxa were polymorphic with respect to all loci included in this study, as evidenced by the allele networks (Fig. 3). None of the nuclear introns show species monophyly, and there are many shared alleles between taxa. In contrast, the mitochondrial CR is much more segregated between the taxa, with only one shared haplotype and very little intermixing – however, two *I. spurius* haplotypes are only one base pair different than the closest *I. fuertesi* haplotype, while they are either two or three base pairs different than the closest *I. spurius* haplotypes.

Both taxa show high levels of haplotype diversity (Hd) for all of the nuclear loci, ranging from 0.724 (SLC9 for *I. spurius)* to 0.889 (TGFB2 for *I. fuertesi*) (Table 2). However, both show lower levels of diversity for the mitochondrial gene CR, with 0.597 for *I. spurius* and 0.242 for *I. fuertesi*. Note that *I. spurius* has more than two times the haplotype diversity of *I.  fuertesi* for CR, yet the taxa show similar amounts of diversity for all four nuclear loci (Table 2). In terms of nucleotide diversity (*π*), three of the five loci (CR, TGFB2, and RDP2) have higher nucleotide diversity for *I. spurius* and two (GADPH11 and SLC9) have higher diversity for *I. fuertesi*.

The majority of the loci for both taxa show negative values for Tajima's *D* and for Fu and Li's F*, with a few exceptions (Table 3). However, none of these values differed significantly from zero once Bonferroni corrections were applied, indicating that there is no evidence of selection within the loci included in this study. The sizes of the Ramos‐Onsins & Rozas *R*_2_ values indicate that both taxa may have undergone recent population expansions (Ramos‐Onsins and Rozas 2002).

**Table 3:** Results of tests for neutrality and constant populations sizes for *Icterus spurius* and *I. fuertesi*

Locus	Tajima's *D*		Fu and Li's *F* *		Ramos‐Onsins and Rozas *R*_2_	
	*I. spurius*	*I. fuertesi*	*I. spurius*	*I. fuertesi*	*I. spurius*	*I. fuertesi*
CR‐ mtDNA	−1.965*	−1.349	−2.271	−1.351	0.054	0.166
GADPH11	−0.046	1.0921	−0.637	1.375	0.101	0.177
TGFB2	−1.276	−1.008	−2.264	−2.098	0.067	0.091
RDP2	−0.052	−0.059	−0.471	0.354	0.109	0.124
SLC9	−1.965	1.014	0.152	1.309	0.087	0.186

Significant value (0.05 > *P *>* *0.01) is indicated by *. None of the below values remained significant after Bonferroni corrections (adjusted at a level of 0.01).

**Figure 4:**
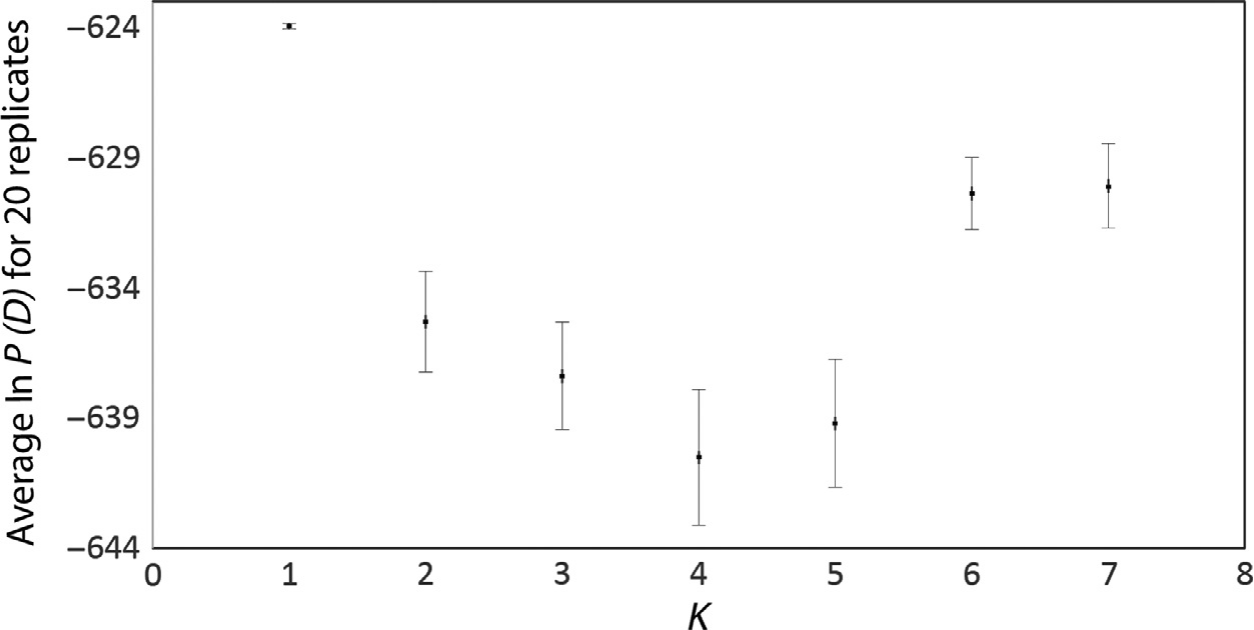
Mean Ln *P*(*D*) with SE for *K* = 1 to 10, with 20 replicates for each *K*, for nuclear loci for *Icterus spurius* and *I. fuertesi* (Structure v. 2.3.4).

**Figure 5:**
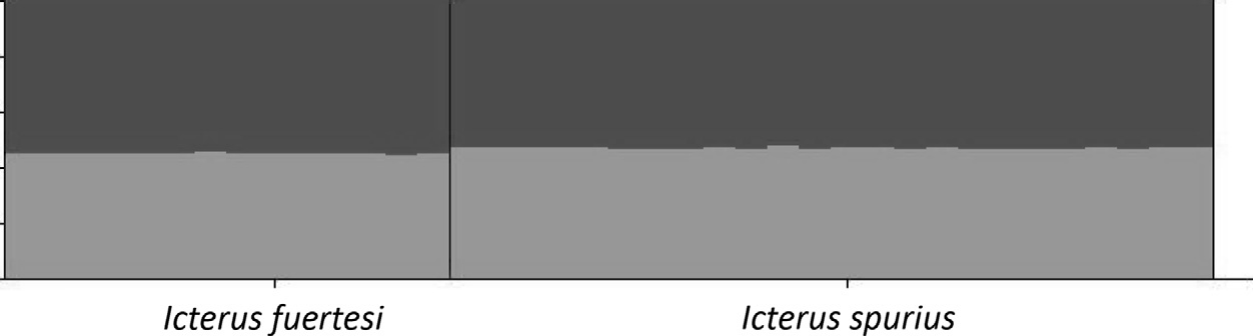
Results of Structure clustering analysis for nuclear loci for *K* = 2 using Structure v. 2.3.4.

The results from STRUCTURE analysis of the four nuclear loci provide no evidence of population structure. The highest log‐likelihood probability for Ln *P*(*D*) was detected for *K* = 1 (Fig. 4), and structure was unable to group individuals into clusters that matched taxon boundaries (Fig. 5). The AMOVA results for these loci generally agree with this finding, as they show that the majority of the haplotype variation can be explained by within population divergence (see Table 4). Thus, there is generally a lack of nuclear intron structure between the two taxa, with a percent of variance explained by species boundaries that ranges from −6.3% (SLC9) to 5.6% (RDP2), see Table 4 for additional values. These findings are further supported by three of the four nuclear loci, which had nonsignificant Φ_ST_ values, with the vast majority of the variation being explained by within population variation. The remaining autosomal locus, TGFB2, however, has an important difference; whereas the majority of variation within this locus is again explained by within population variation, TGFB2's Φ_ST_ value was significant, with a value of 0.056 (*P *<* *0.00001).

**Table 4:** AMOVA results and ɸ_ST_ values for all loci included in this study

Locus	Φ_ST_	Percentage of variation among species	Percentage of variation among populations within species	Percentage variation within populations
CR‐ mtDNA	0.717**	71.3	1.1	27.7
GADPH11	−0.009	1.2	−6.9	105.8
TGFB2	0.056**	5.1	1.3	93.6
RDP2	0.044	5.6	−2.9	97.4
SLC9	0.086	−6.3	22.1	84.2

Significance value (*P *<* *0.01) indicated by ** (*P*‐values Bonferroni corrected).

For the IM analyses of our nuclear loci, the IM runs consistently failed to converge even after more than twenty runs with the autosomal loci – the last five of which included the Z‐linked intron SLC9 – and using multiple random number seeds. (The failure to converge could be caused by recent divergence; other recent publications from our laboratory used similar methods and resulted in convergence; see Discussion). As a result, we will not present the results from our IM analyses in this article.

The results of the AMOVA for mtDNA CR are a clear contrast to the nuclear DNA (Table 4). These results show statistically significant structure between the two taxa, with 71.3% of genetic variation being explained by species boundaries. Additionally, the Φ_ST_ value for this locus is 0.717 (*P *<* *0.00001), indicating there is evidence of a great deal of structure between the two taxa for this locus. With the addition of more *I. fuertesi*, the mtDNA haplotype frequencies are clearly statistically significantly different.

## Discussion

### Do nuclear DNA support very recent divergence?

The majority of the nuclear loci included in this study show very little evidence of structure between the two taxa. One nuclear locus, TGFB2, is the exception as it has a small, but significant Φ_ST_ value (*Φ*_*ST*_ = 0.056, *P *<* *0.00001). The amount of variation explained at the among‐species level in the AMOVA for TGFB2 is 5.1%, indicating that a small portion of the genetic variation can be explained by species boundaries. If the mtDNA paraphyly first described by Baker et al. (2003) was the result of a recent mitochondrial introgression between two well‐diverged lineages, we would expect to find a great deal of divergence between the nuDNA loci that would not be reflected in the mtDNA (Jacobsen and Omland 2012). Instead, these two lineages appear to be very early in the process of diverging – as evidenced by the lack of structure for the majority of nuclear loci, as well as by the lack of reciprocal monophyly in mtDNA.

With increased sampling, we found significant differences in mitochondrial haplotype frequencies between the two populations that confirms gene flow between the two taxa may be restricted (Baker et al. 2003). For the mitochondrial gene CR, there is clear evidence of significant population structure between the two taxa, with a Φ_ST_ value of 0.717 (*P *<* *0.00001). Even with the inclusion of eight more *I*. *fuertesi* samples, only one haplotype is shared between the two taxa for this locus, and only two haplotypes are intermixed and showing mtDNA paraphyly. Two haplotypes that we found only in *I. spurius* are only one base pair different from *I. fuertesi* haplotypes (Fig. 3, CR), yet are at least two base pairs different than the closest *I. spurius* haplotypes. Thus, with increased sampling effort, CR seems to be nearing reciprocal monophyly between groups. These lineages show a classic pattern of intermediate divergence in which the only haplotype shared between the taxa is a central, likely ancestral haplotype. Omland et al. (2006) discussed this as an early step on the road to monophyly (“neotypy”) indicating that these two taxa are clearly showing evidence of divergence within the faster sorting mtDNA.

### Does nuclear DNA support two distinct groups?

In comparison, the nuclear DNA included in this study show very little evidence of structure between the two taxa. The IM analyses failed to converge even though we tried a range of strategies to produce convergence. We have published a number of recent studies from our laboratory on other species of recently diverged *Icterus* using similar methods, which resulted in convergence in those cases (Jacobsen and Omland 2012; Cortés‐Rodríguez et al. 2013; Cortés‐Rodríguez and Omland 2016). Furthermore, in the Orchard species complex, all four nuclear loci show a great deal of intermixing of alleles (Fig. 3). Additionally, the results of the AMOVAs indicates that almost all of the variation is found within the populations of each taxon (Table 4), further supporting a lack of divergence within the slower sorting nuclear loci included in the study. This is what we would expect to find if the divergence between these two taxa was at the very earliest stages of speciation.

Although the majority of variation within the nuclear loci exists at the level of variation within populations, one autosomal locus showed a small amount of variation that could be explained by species boundaries. TGFB2 had roughly 5% of its variation being explained by variation within each taxon (*P *<* *0.00001). Thus, one of the nuclear loci shows some evidence of segregation, indicating that the nuclear genomes of the two taxa are beginning to diverge.

Our inability to find much structure for the nuclear loci does not necessarily mean the taxa are not evolutionarily discrete units. There are many examples of known mitochondrial paraphyly within ornithological literature, with a large portion of it being attributed to retained ancestral polymorphisms (Funk and Omland 2003; Mckay and Zink 2010). Neutral nuclear markers, with their larger ESSs and slower sorting rates, are predicted to lag even further behind (Price 2008; Zink and Barrowclough 2008; Joseph and Omland 2009). Taxa that fall into this category may still be discrete in other ways that indicate they are evolutionarily distinct units, for example, for traits under sexual selection such as song or plumage coloration (Edwards et al. 2005; Marthinsen et al. 2008; Price 2008; Joseph and Omland 2009; Mckay and Zink 2010). A study examining a recent divergence in two species of crows, *Corvus (corone) corone* and *C. (corone) cornix*, showed significant divergence in the genes that controlled for plumage coloration of the two taxa, yet a lack of divergence in neutral genetic markers due to hybridization and widespread introgression, showing that assortative mating and sexual selection can lead to genetic differentiation in regions under selection (Polestra et al. 2014). Similar to the above study, research in our laboratory has previously shown that *I. spurius* and *I. fuertesi* have fixed differences in adult male coloration (Hofmann et al. 2007; Kiere et al. 2007). In a parallel study to this one, we found fixed differences between the taxa in a call that seems to play a role in territory defense, thus might be under the influence of sexual selection (Sturge et al. 2016). Any genes that could be contributing to either plumage coloration or this call thus have the potential to have diverged between these two taxa.

### Did *I. fuertesi* result from a founder event?

If the divergence event that separated *I. fuertesi* and *I. spurius* resulted from a few individuals of the migratory common ancestor founding a new, nonmigratory or short‐distance migratory population, followed by peripheral isolates speciation (Mayr 1942, 1963; West‐Eberhard 2005), we would predict that *I. fuertesi* would have significantly less genetic diversity than *I. spurius*. Looking at both the haplotype networks (Fig. 3) and the population statistics for each locus (Table 2), it is clear that both *I. spurius* and *I. fuertesi* have similar levels of genetic diversity for all nuclear loci included in this study (although the mitochondrial locus still indicates less genetic diversity within *I. fuertesi*). Based on these results, we found no evidence of a founder event in *I. fuertesi'*s recent past. This result is surprising because the distribution of *I. fuertesi* suggested that it might have evolved through this mechanism. Instead, both taxa show a great deal of allelic diversity, indicating relatively large historic population sizes in spite of the very restricted range of *I. fuertesi*. Nevertheless, it is conceivable that *I. fuertesi* was established through a large number of founders, and/or that subsequent gene flow from *I. spurius* has added a lot of genetic diversity through introgression. As the IM analyses did not converge, we are unable to completely eliminate this possibility without including many additional loci. However, a classic splitting of a single more widespread population clearly fits our data. This taxon pair would be ideally suited to next generation sequencing approaches to study this and other aspects of their divergence (e.g., testing for genes affecting plumage coloration and ecological tolerances). Whole genome comparisons would most likely provide more insight into both the divergence between these two taxa, and any potential gene flow that has occurred since this divergence. The statistical results of the Ramos‐Onsins and Rozas R_2_ for both taxa (Table 3) does seem to support recent and rapid population expansion for both taxa, which may have contributed to problems with IM (Hey and Nielsen 2007). Nevertheless, both taxa also have a great deal of both nucleotide and haplotype diversity, as well as unique haplotypes, supporting the existence of large historic ESSs.

### Is it possible to determine whether the two taxa are currently exchanging genes?

Based on the lack of convergence in IM, due largely to the lack of structure within the nuclear loci, we are unable to determine how much of the shared variation is the result of retained ancestral polymorphisms or is the result of gene flow. The control region shows far more evidence of structure, with only one shared haplotype and two intermixed haplotypes within all individuals included in this study. There are two possible explanations: (1) significantly different haplotype frequencies in mtDNA between the taxa indicates gene flow is likely greatly reduced or nonexistent and is not being captured at the nuDNA level due to retained ancestral polymorphisms, or (2) haplotype frequency differences for only the mtDNA could also be explained by Haldane's rule in the face of ongoing gene flow (Mckay and Zink 2010; Peters et al. 2012). Haldane's rule predicts that hybrids of the heterogametic sex will have reduced fitness or be sterile – so that mtDNA in birds would be less likely to introgress from one species to another (Coyne 1985; Peters et al. 2012). As a consequence of this, as well as the failure of the IM analyses to converge, it is difficult if not impossible to determine whether gene flow is currently occurring between these two taxa, or has historically occurred since their divergence. Although their populations breed in allopatry, gene flow could occur as migratory *I. spurius* pass through *I. fuertesi*'s breeding range in early spring. However, the significant population structure, not only within the mtDNA (which is less likely to introgress), but also within the nuclear locus TGFB2, indicates that *I. spurius* and *I. fuertesi* are in the process of diverging genetically from one another. Based on this and other evidence such as coloration and migratory patterns, it is likely that these two taxa have become evolutionarily distinct groups, regardless of any potential gene flow.

## Conclusions – Model Example of Early Divergence

*Icterus spurius* and *I. fuertesi* are two taxa that are in the very earliest stages of speciation. Their mtDNA show only a few shared and intermixed haplotypes, having almost achieved reciprocal monophyly for this locus. The four nuclear loci included in this study show very little population structure between the two taxa – yet one locus (TGFB2) shows a small, but significant Φ_ST_ divergence between the two taxa, confirming the formation of two distinct genome pools. Based on these molecular findings, as well as the fixed differences in plumage, migratory behavior, and bioclimatic niches, there is strong evidence that these two taxa are just beginning to diverge from one another, making them ideal study organisms for the early stages of speciation and the formation of species boundaries (Hofmann et al. 2007; Kiere et al. 2007; Kondo and Omland 2007; Martin and Omland 2011).

## Conflict of Interest

None declared.

## Supporting information


**Table S1.** Specimen numbers and sampling locations for tissue samples.
