# Supplementary material for: Nuclear locus divergence at the early stages of speciation in the Orchard Oriole complex
Source: Ecol Evol. 2016 May 30;6(13):4307–17. doi: 10.1002/ece3.2168 (PMC4930982; doi:10.1002/ece3.2168)
Supplement: Supplementary file 1 — Table S1. Specimen numbers and sampling locations for tissue samples. [file ECE3-6-4307-s001.docx]

**Supplementary Table: Specimen numbers and sampling locations for tissue samples.**

| **Museum Voucher** | **Species** | **Location Collected** |
| --- | --- | --- |
| MMNH 42538 | *Icterus fuertesi* | Tlacotalpan, Veracruz |
| MZFC 22636 | *Icterus fuertesi* | Tlacotalpan, Veracruz |
| MZFC 27067 | *Icterus fuertesi* | Tlacotalpan, Veracruz |
| MZFC 22637 | *Icterus fuertesi* | Tlacotalpan, Veracruz |
| MZFC 22632 | *Icterus fuertesi* | Tlacotalpan, Veracruz |
| MZFC 22631 | *Icterus fuertesi* | Las Barrancas, Veracruz |
| MZFC 22634 | *Icterus fuertesi* | Las Barrancas, Veracruz |
| MZFC 22633 | *Icterus fuertesi* | Las Barrancas, Veracruz |
| MZFC 22635 | *Icterus fuertesi* | Las Barrancas, Veracruz |
| MZFC 22638 | *Icterus fuertesi* | Las Barrancas, Veracruz |
| MZFC 15535 | *Icterus fuertesi* | Tecolutla, Veracruz |
| MZFC 15534 | *Icterus fuertesi* | Tecolutla, Veracruz |
| MZFC 15533 | *Icterus fuertesi* | Tecolutla, Veracruz |
| MZFC 15532 | *Icterus fuertesi* | Tecolutla, Veracruz |
| MZFC 5439 | *Icterus spurius* | Santiaguillo, Guanajuato |
| MZFC 3474 | *Icterus spurius* | San Pedro d.l. Naranjos, Guanajuato |
| MZFC 5440 | *Icterus spurius* | Santiaguillo, Guanajuato |
| MZFC 5454 | *Icterus spurius* | Santiaguillo, Guanajuato |
| LSUMZ B-5929 | *Icterus spurius* | Cameron Parish, LA |
| LSUMZ B-6377 | *Icterus spurius* | Cameron Parish, LA |
| LSUMZ B-6378 | *Icterus spurius* | Cameron Parish, LA |
| LSUMZ B-6379 | *Icterus spurius* | Cameron Parish, LA |
| LSUMZ B-6380 | *Icterus spurius* | Cameron Parish, LA |
| LSUMZ B-6382 | *Icterus spurius* | Cameron Parish, LA |
| LSUMZ B-8464 | *Icterus spurius* | Cameron Parish, LA |
| USNM 626504 | *Icterus spurius* | Okaloosa, FL |
| NCSM 15229 | *Icterus spurius* | Pender County, NC |
| UKNHM 91051 | *Icterus spurius* | Seward County, KS |
| ANSP 10210 | *Icterus spurius* | Accomack County, VA |
| USNM 633857 | *Icterus spurius* | Lee County, VA |
| ANSP 10125 | *Icterus spurius* | Bucks County, PA |
| ASNP 10147 | *Icterus spurius* | Bucks County, PA |
| FMNH 381975 | *Icterus spurius* | Chicago, IL |
| FMNH 383513 | *Icterus spurius* | Chicago, IL |
| FMNH 383514 | *Icterus spurius* | Chicago, IL |
| FMNH 389579 | *Icterus spurius* | Chicago, IL |
| FMNH 390351 | *Icterus spurius* | Lisle, IL |
| FMNH 394644 | *Icterus spurius* | Chicago, IL |
| LSUMZ B-3980 | *Icterus spurius* | Weld County, CO |
